# Caerulein-induced acute pancreatitis in mice that constitutively overexpress *Reg/PAP *genes

**DOI:** 10.1186/1471-230X-6-16

**Published:** 2006-05-15

**Authors:** Oxana Norkina, Rolf Graf, Philippe Appenzeller, Robert C De Lisle

**Affiliations:** 1 Anatomy and Cell Biology, University of Kansas School of Medicine, Kansas City, Kansas, USA; 2 Pancreatitis Research Laboratory, Division of Visceral and Transplantation Surgery, University Hospital, Zurich, Switzerland

## Abstract

**Background:**

The cystic fibrosis (CF) mouse pancreas has constitutively elevated expression of the *Reg/PAP *cell stress genes (60-fold greater *Reg3α*, and 10-fold greater *PAP/Reg3β *and *Reg3γ*). These genes are suggested to be involved in protection or recovery from pancreatic injury.

**Methods:**

To test this idea the supramaximal caerulein model was used to induce acute pancreatitis in wild type and CF mice. Serum amylase, pancreatic water content (as a measure of edema), pancreatic myeloperoxidase activity, and *Reg/PAP *expression were quantified.

**Results:**

In both wild type and CF mice caerulein induced similar elevations in serum amylase (maximal at 12 h), pancreatic edema (maximal at 7 h), and pancreatic myeloperoxidase activity (MPO, a marker of neutrophil infiltration; maximal at 7 h). By immunohistochemistry, Reg3α was strongly expressed in the untreated CF pancreas but not in wild type. During pancreatitis, Reg3α was intensely expressed in foci of inflamed tissue in both wild type and CF.

**Conclusion:**

These data demonstrate that the severity of caerulein-induced pancreatitis is not ameliorated in the CF mouse even though the *Reg/PAP *stress genes are already highly upregulated. While Reg/PAP may be protective they may also have a negative effect during pancreatitis due to their anti-apoptotic activity, which has been shown to increase the severity of pancreatitis.

## Background

There is a strong association of the *Reg/PAP *genes with pancreatic stress and injury, especially in response to pancreatitis [1,2]. The role of these proteins has been investigated under various conditions. PAP appears to have an anti-inflammatory effect in pancreatic injury [3] as well as in inflammatory bowel disease [4]. In vitro experiments demonstrated that PAP can inhibit TNF-α mediated inflammatory responses of macrophages [3] and of epithelial and endothelial cells [4]. Experimental evidence also suggests that Reg/PAP are mitogenic and/or anti-apoptotic and enhance cell survival during development and in injured tissues [5-7]. The anti-apoptotic activity of Reg/PAP is of interest to pancreatitis, as experimental evidence shows that reduction of apoptosis can be associated with a worsened severity of pancreatitis [8,9].

The Reg/PAP proteins are synthesized in a soluble form that, upon tryptic cleavage of an 11 amino acid N-terminal fragment, undergo conversion to fibrils [10]. It has been proposed that the fibrils could form clot-like structures which, intracellularly would help control cell damage, and extracelluarly would preserve the integrity of the ductal epithelium during pancreatitis [11].

Because there are seven known *Reg/PAP *genes which span about 75 kb of mouse chromosome 6 [12] plus *Reg4 *on chromosome 3 [13], it has not been practical to knockout all these genes together to study their functions. Although one knockout model exists for *Reg1/PSP *(pancreatic stone protein) [5], the presence of multiple isoforms of PAP (*Reg3*) may allow compensation for the loss of a single form and result in the potential absence of a phenotype. Therefore, we took advantage of the *CFTR *(cystic fibrosis transmembrane conductance regulator) null mouse (CF mouse), which has constitutively elevated expression levels of the *Reg3α*, *PAP/Reg3β *and *Reg3γ *genes [14]. CFTR is a cAMP-activated chloride channel expressed in various epithelia of the body, and is especially important for fluid and bicarbonate ion secretion in the gastrointestinal system to neutralize gastric acid in the small intestine [15]. In humans, the pancreas depends on CFTR for fluid and bicarbonate ion secretion and it is one of the most strongly damaged organs in CF when CFTR is absent or nonfunctional due to mutation [16]. In contrast, the CF mouse pancreas is only mildly affected by loss of CFTR function [9,17,18]. A likely reason for this is that the mouse expresses a calcium-regulated chloride channel and the mouse pancreatic duct is not reliant on CFTR for proper function [19,20]. A secondary effect of CF caused by the most common CFTR mutation, ΔF508, may be due to misfolded CFTR protein and subsequent activation of the endoplasmic reticulum unfolded protein response [21]. This secondary effect of CF is absent in the mouse model as there is no *Cftr *mRNA or protein expressed in the null mouse [22].

To explain why there were changes in gene expression in the CF mouse pancreas, despite the fact that the mouse ductal system does not rely on CFTR for proper function, it was hypothesized that another CF-affected organ could be involved [14]. It has been demonstrated that the luminal pH of the CF mouse duodenum is abnormally acidic [14,23], and evidence was presented showing excess cAMP-mediated signaling by the small intestine to the pancreas, in an attempt to stimulate more bicarbonate ion secretion [24]. It is known that cAMP potentiates calcium-mediated signaling in the acinar cell, and a chronically elevated cAMP signal would be expected to increase the secretory activity of the acinar cell. Chronic stimulation of the acinar cell could result in altered gene expression as an adaptive response to this stress on the secretory pathway. This idea was supported by the finding that pharmacological or genetic correction of the duodenal pH in CF mice largely reversed the overexpression of *Reg/PAP *genes [14].

For these reasons, the CF mouse pancreas presents a useful system to investigate the effects of *Reg/PAP *gene expression on the severity of pancreatitis. We postulated that in the CF mouse pancreas the constitutively elevated expression of *Reg/PAP *genes would lead to a less severe course of experimentally-induced acute pancreatitis. Contrary to the hypothesis, the results show that caerulein-induced pancreatitis is similar in CF mice despite high levels of expression of *Reg/PAP *genes.

## Methods

### Animals

CFTR(+/-) mice (*cftr*^*tm1UNC*^) were obtained from Jackson Labs (Bar Harbor, ME, USA) and have been backcrossed on the C57Bl/6 background until congenic as previously described [24]. CFTR(+/-) mice were bred to obtain wild type [CFTR(+/+)] and CF [CFTR(-/-)] mice. Mice of both sexes were used between 6–7 weeks of age and no gender-related differences in the measured parameters were observed. To prevent lethal intestinal obstruction, CF mice were fed ad libitum a complete elemental liquid diet (Peptamen; Nestle, Deerfield, IL, USA) [25] and wild type were fed the same diet to avoid potential dietary effects. All procedures were approved by the University of Kansas Medical Center Institutional Animal Care and Use Committee.

### Caerulein-induced acute pancreatitis

Pancreatitis was induced by intraperitoneal injection of 50 μg/kg caerulein (Sigma, St. Louis, MO, USA) in 0.9% NaCl [26]. Controls were untreated or received equal volumes of 0.9% NaCl injected intraperitoneally. There were no differences between untreated and NaCl injected mice for any parameters measured, so they were grouped together for analysis. Samples were taken from mice 1 h after a single caerulein injection, and from mice at 7 h, 12 h, 3 days, and 7 days after the beginning of 7 caerulein injections at hourly intervals.

### Analysis of acute pancreatitis

Animals were sacrificed with CO_2 _gas and trunk blood was collected for serum preparation. Serum amylase was determined using 4,6-ethyldiene(glucose)_7_-*p*-nitrophenyl-glucose-α, D-maltoheptaside (Raichem, San Diego, CA, USA). The pancreas was removed and a small portion was taken for preparation of total RNA using the Trizol reagent (Invitrogen, Carlsbad, CA, USA) following the supplier's instructions with modifications to enhance the integrity of pancreatic RNA. First, it was determined that immediate homogenization of the tissue in Trizol was required for isolation of high quality RNA, and that freezing of the tissue in liquid nitrogen followed by later processing was not effective at stabilizing the RNA. Second, a number of modifications to the isolation procedure were made. The Trizol was prechilled and homogenization was performed on ice using a 100:1 ratio of Trizol to tissue instead of the 10:1 ratio the instruction manual suggests. Pancreatic RNA was stable in the Trizol homogenates stored at -70°C until processing. To isolate total RNA, the following steps were used: (i) thaw sample on ice and centrifuge 12 k × g × 10 minutes at 4°C; (ii) transfer the supernatant to a fresh tube, add 200 μl chloroform, mix well by inversion for 15 sec, and incubate 2 minutes at room temperature; (iii) centrifuge 12 k × g × 15 min at 4°C; (iii) transfer the upper phase to a fresh tube, add 0.5 ml isopropanol, mix by inversion and vortexing, and incubate at room temperature for 10 min; (iv) centrifuge 10 k × g × 10 min at 4°C; (v) discard the supernatant and resuspend the pellet in 1 ml 75% ethanol; (vi) centrifuge at 7.5 k × g × 5 min at 4°C; (vii) remove supernatant, dry under vacuum, and resuspend pellets in diethylpyrocarbonate-treated water.

Another piece of tissue was fixed overnight in 4% paraformaldehyde in phosphate-buffered saline for histology; and the remaining small piece was blotted to remove excess buffer and weighed, and rapidly frozen in liquid nitrogen. The frozen tissue was lyophilized to dryness (3 days) and weighed again to determine percent wet weight as a measure of tissue edema. The dried pancreas samples were homogenized and extracted with hexadecyltrimethylammonium-bromide followed by determination of myeloperoxidase (MPO) activity as an estimate of neutrophil infiltration as described [27].

### Analysis of gene expression

Total RNA prepared from the pancreas was used to measure gene expression as previously described [14]. Briefly, real time RT-PCR was performed on an iCycler (Bio-Rad, Hercules, CA, USA) with a one-step RT-PCR kit (Qiagen, Valencia, CA, USA) that employs the double stranded DNA-binding dye SYBR Green. Serial dilutions of cloned plasmid DNAs for the genes of interest were used to generate standard curves. The genes measured were: *Reg3α *(GenBank accession NM_011259), *PAP/Reg3β *(NM_011036), and *Reg3γ *(NM_011260). Levels of 18S rRNA measured by quantitative RT-PCR and total RNA measured by OD_260 nm _were used for normalization. Because there were changes in levels of 18S rRNA (see Results), data are expressed as copies of specific mRNAs per μg total RNA.

### Production of recombinant Reg3α and development of antibodies

For the preparation of cDNA coding for mouse Reg3α(GenBank accession NM_011259), mRNA was extracted and reverse transcribed from mouse pancreas as described [28]. The cDNA was amplified by PCR using a forward primer (5'-GAA GAA GGG GTA TCT CTC GAG AAA AGA **CAA GGT GAA GAC TTC CAG AAG G**-3') that includes an XhoI restriction site followed by a sequence coding for the Kex2 cleavage site and a Reg3α specific sequence at the 3'-end (shown in bold). The reverse primer (5'-CTA CTG CTT GAA CTT GCA GAC-3) annealed to the 3'-untranslated region of the cDNA. The cDNA was subcloned into pcR2.1-TOPO (Invitrogen) and sequenced to verify the identity of the gene. The cDNA was then excised from the plasmid by restriction digestion with XhoI and NotI and subcloned into pPIC9. The plasmid was then transformed into *Pichia pastoris *strain KM71 and recombinant protein was generated and purified as described [28]. The recombinant protein consisted of amino acid 25 through the end of Reg3α and was used to raise rabbit antisera. An ELISA for Reg3α was then established using antibodies generated in rabbits and guinea pigs as described previously [28,29]. Paraffin sections (5 μm) of pancreas were processed for immunohistochemistry of Reg3α using the Vectastain ABC technique (Vector Labs, Burlingame, CA, USA).

### Statistics

Comparisons were made with Systat software (Systat, Chicago, IL, USA). Initial analysis showed that the values were not normally distributed, so the nonparametric Kruskal-Wallis one-way analysis of variance was used. *P *values of less than 0.05 were considered significant. Values are plotted as means and sem.

## Results

**Figure 1:**
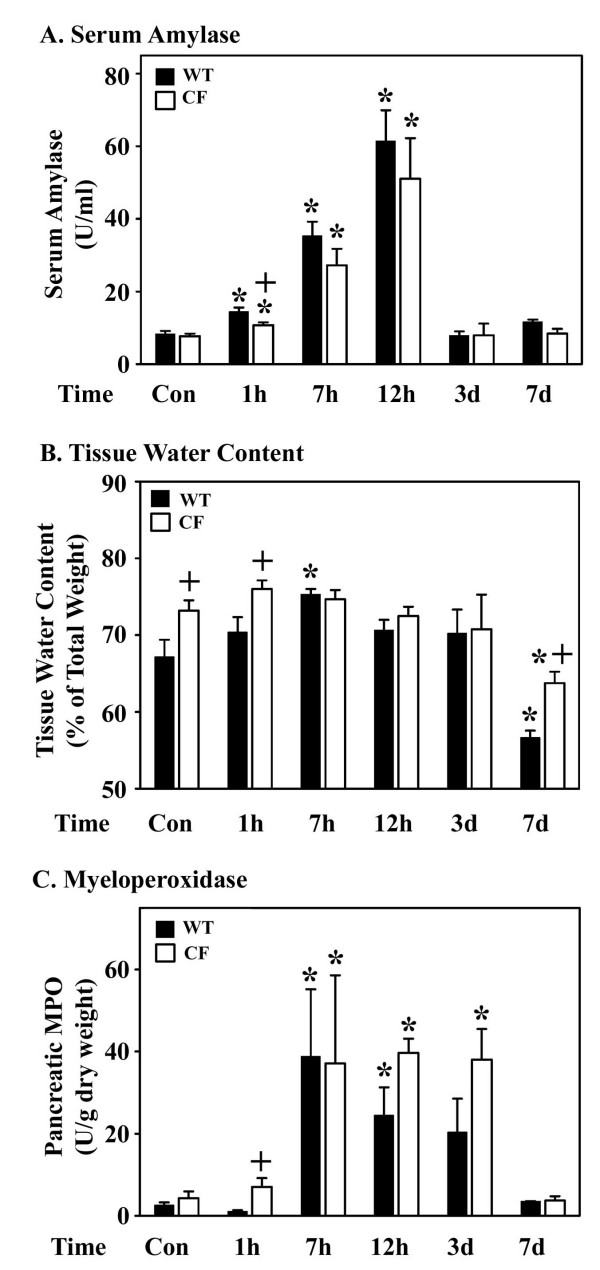
**Severity of acute pancreatitis in wild type and CF mice**. (A) Serum amylase. Trunk blood was collected at sacrifice for measurement of serum amylase activity. (B) Tissue water content as a measurement of edema. Pancreatic tissue was obtained at sacrifice and the wet weight determined. The tissue was then lyophilized to dryness and the dry weight was determined. Data are expressed as the water content of the tissue (% wet weight). (C) Pancreatic myeloperoxidase (MPO) activity. Pancreatic tissue was obtained at sacrifice and was then lyophilized to dryness. The tissue was processed and myeloperoxidase (MPO) activity was determined as described in the Materials and Methods. Data are expressed units per g dry weight of the tissue. For (A-C) control mice were untreated or injected with 0.9% NaCl. Mice for the 1 h time point received a single injection of caerulein and were sacrificed one hour after injection. The other data are from mice that received 7 hourly caerulein injections and the times are relative to the first injection. The data are means and sem. (*) *p *< 0.05 compared to control of the same genotype. (+) *p *< 0.05 comparing CF to wild type at the same time point. n = 3 to 7 mice per group and time point.

To test whether constitutive elevation of *Reg/PAP *genes in the CF mouse pancreas affects the severity of acute pancreatitis, the caerulein supramaximal stimulation model was used [30]. We measured parameters of acute pancreatitis after a single caerulein injection as well as at different times after a series of seven hourly injections. As previously reported [24], there was no difference in serum amylase comparing control wild type and CF mice (Fig. 1A). Serum amylase levels were significantly increased in both wild type and CF mice at 1 h after a single injection as well as 7–12 h after the first of 7 hourly injections of caerulein. Serum amylase levels recovered to control values by 3 days in both genotypes of mice (Fig. 1A). There was slightly less serum amylase activity in CF mice compared to wild type after a single caerulein injection and at 7 h and 12 h after the complete series of caerulein injections (Fig. 1A) which may be related to the decreased tissue content of digestive enzymes in the CF mouse pancreas (see Discussion).

Pancreatitis is accompanied by tissue edema which was measured by determining the water content of the tissue. In the wild type mouse pancreatic edema was maximal at 7 h (Fig. 1B). In the control CF pancreas the tissue water content was already slightly greater than wild type (Fig. 1B). The water content of the CF pancreas was greater after a single caerulein injection and at 7 d, after resolution of pancreatitis, compared to the corresponding wild type tissue (Fig. 1B).

Injury to acinar cells in pancreatitis causes release of cytokines and recruits neutrophils which can be measured as an increase in tissue myeloperoxidase (MPO) activity [31]. As shown in Fig. 1C, control wild type and control CF mice both have low pancreatic MPO levels. One hour after a single caerulein injection the MPO level in the CF tissue was slightly increased over control while the wild type level was not yet changed (Fig. 1C). Between 7–12 h after caerulein injections, there were significant increases in MPO activity in both wild type and CF pancreas (Fig. 1C). The elevated MPO levels persisted longer in the CF mice, remaining significantly increased at 3d after caerulein administration (Fig. 1C). In both wild type and CF mice, there was a return to control levels by 7d after caerulein injection.

**Figure 2:**
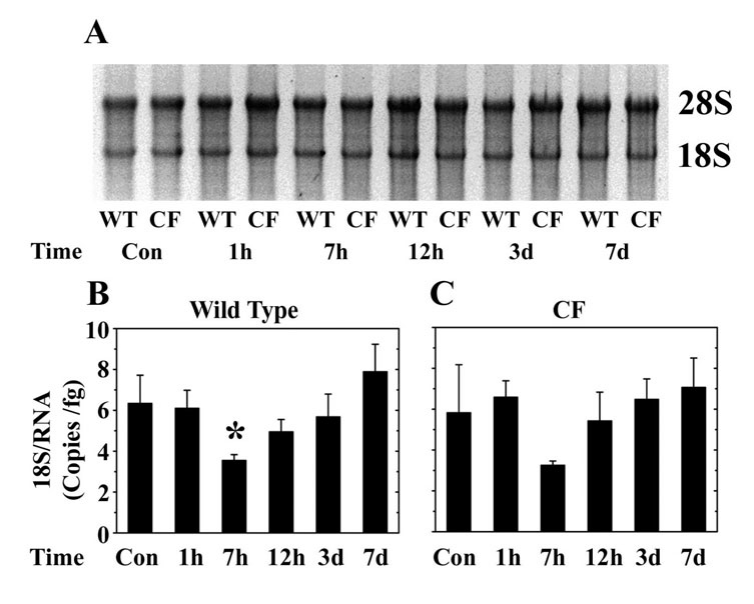
**Assessment of RNA quality and levels of 18S RNA in wild type and CF mice during acute pancreatitis**. (A) RNA quality. Five micrograms of total RNA were loaded per lane and electrophoresed, the gel was stained with SYBR Green II, and imaged with a Bio-Rad ChemiDoc XRS digital system. Representative of duplicate samples. The time points indicated refer to the elapsed time after the first caerulein injection. (B, C) qRT-PCR quantification of 18S RNA per total RNA. (B) Wild type pancreas samples. (C) CF pancreas samples. The data are means and sem. (*) *p *< 0.05 compared to control of the same genotype. n = 3 to 7 mice per group and time point.

Using quantitative real-time RT-PCR (qRT-PCR), we next examined expression of the *Reg/PAP *genes that are associated with pancreatitis [1], and the mild inflammation of the CF mouse pancreas [14]. For normalization of qRT-PCR data 18S rRNA and total RNA values were considered. Total RNA was intact and of high quality as assessed by gel electrophoresis (Fig. 2A). By densitometry, ratios of 28S to 18S rRNAs were about 2 and did not vary with caerulein treatment nor genotype. Using qRT-PCR to measure 18S per fg of total RNA, there was a decrease in 18S at the 7 h time point in both wild type and CF pancreas (Fig. 2B and 2C, respectively). Therefore, mRNA values for the genes of interest were expressed relative to total RNA.

**Figure 3:**
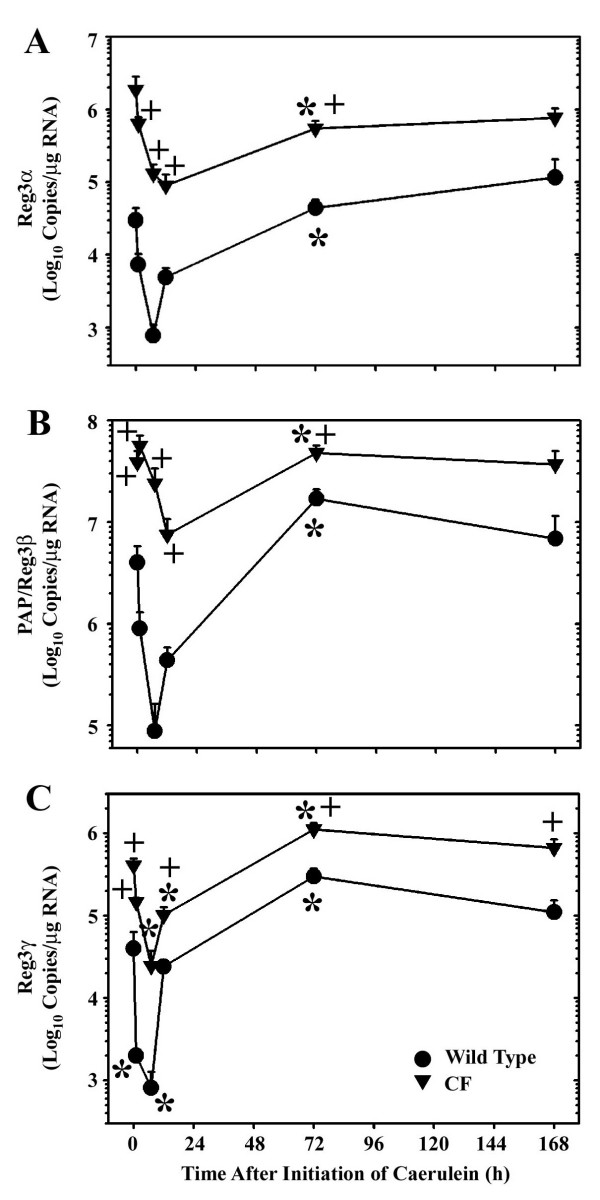
**Pancreatic *Reg/PAP *gene expression in wild type and CF mice during acute pancreatitis**. Pancreatic tissue was obtained at sacrifice and processed to extract total RNA as described in the Materials and Methods. Gene expression levels were determined by real time quantitative RT-PCR. (A) *Reg3α*; (B) *PAP/Reg3β*; (C) *Reg3γ*. Data are expressed as copies of gene specific mRNA per μg total RNA. Control mice were untreated or injected with 0.9% NaCl. Mice for the 1 h time point received a single injection of caerulein and were sacrificed one hour after injection. The other data are from mice that received 7 hourly caerulein injections and the times are relative to the first injection. Values are presented on semi-log plots as means and sem. (*) *p *< 0.05 compared to control of the same genotype. (+) *p *< 0.05 comparing CF to wild type at the same time point. n = 3 to 7 mice per group and time point.

As previously shown, the *Reg/PAP *genes *Reg3α*, *PAP/Reg3β*, and *Reg3γ*, are all significantly elevated in the control CF pancreas compared to wild type (Fig. 3) [14]. The *Reg/PAP *genes had somewhat different time courses of altered expression in caerulein-induced pancreatitis, and there were differences between wild type and CF pancreas. In wild type mice after supramaximal caerulein administration, these genes were transiently downregulated at early times of pancreatitis and increased at later times (Fig. 3). *Reg3α *was down-regulated with a minimum of expression at 7 h, followed by an increase which was maximal at 7d (Fig. 3A). *PAP/Reg3β *showed decreased expression after caerulein treatment, reaching a minimum at 7 h after injection, and exhibiting significant overexpression at 3d (Fig. 3B) consistent with previous work [32]. *Reg3γ *showed a decrease in expression at 1 h and 7 h, and then exhibited strong overexpression with a maximum at 3d after caerulein treatment (Fig. 3C).

In the CF pancreas, the *Reg/PAP *genes also exhibited decreased expression after induction of pancreatitis, and the minima were at 7–12 h for all three genes (Fig. 3). Only *Reg3γ *was significantly overexpressed relative to CF control levels, and this occurred at 3d after caerulein administration (Fig. 3C).

**Figure 4:**
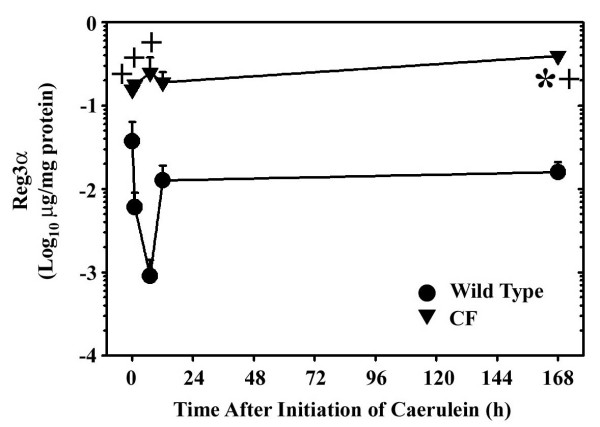
**Quantitation of Reg3α protein in wild type and CF mice during acute pancreatitis**. Pancreatic homogenates were used to measure Reg3α tissue content by ELISA. Values were normalized to total protein content of the homogenates. Control mice were untreated or injected with 0.9% NaCl. Mice for the 1 h time point received a single injection of caerulein and were sacrificed one hour after injection. The other data are from mice that received 7 hourly caerulein injections and the times are relative to the first injection. Values are presented on a semi-log plot as means and sem. (*) *p *< 0.05 compared to control of the same genotype. (+) *p *< 0.05 comparing CF to wild type at the same time point. n = 3 to 7 mice per group and time point.

To determine protein levels of murine Reg3α, an ELISA was used. In wild type mice induction of pancreatitis transiently reduced the protein amounts of Reg3α (Fig. 4) similar to the changes in mRNA levels (Fig. 3A). In the control CF pancreas, Reg3α protein content was significantly greater than wild type control (Fig. 4) but the difference in protein levels was not as great as that for mRNA levels. When pancreatitis was induced in CF mice the levels of Reg3α protein changed little (Fig. 4), in contrast to more substantial changes in mRNA (Fig. 3A).

**Figure 5:**
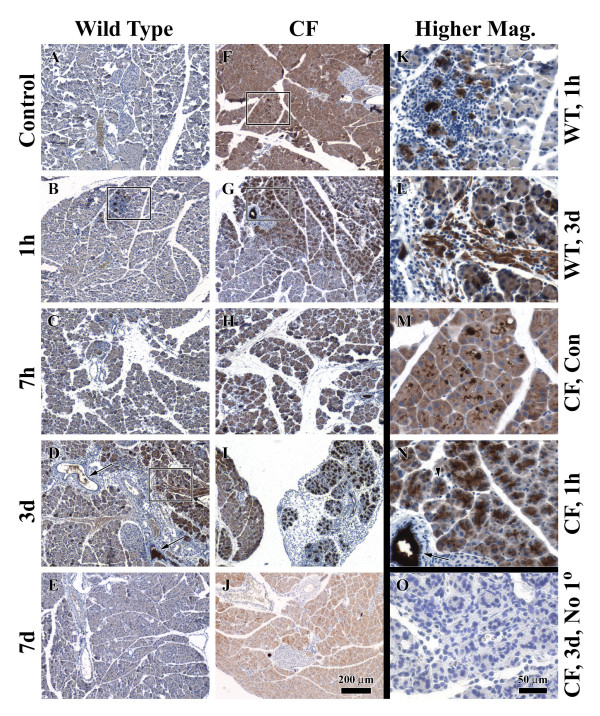
**Immunohistochemistry for Reg3α in wild type and CF mice during acute pancreatitis**. Paraffin sections of pancreas from wild type and CF mice, untreated controls or with caerulein-induced pancreatitis, were stained with a Reg3α-specific antibody by the Vectastain ABC technique. Wild type: (A) untreated control; (B) 1 h after a single injection; (C) 7 h after the beginning of seven injections; (D) 3d after the beginning of seven injections. Note reactivity in duct lumina (arrows); and (E) 7d after the beginning of seven injections. CF: (F) untreated control; (G) 1 h; (H) 7 h; (I) 3d; and (J) 7d. Higher magnification images: (K) wild type, 1 h, from the boxed region in panel (B) showing strong expression in a region of inflammation; (L) wild type, 3d, from the boxed region in panel (D) showing expression in a region of fibrosis; (M) untreated CF, from the boxed region in panel (F) showing strong expression in dilated acinar lumina; (N) CF, 1 h, from the boxed region in panel (G) showing expression in acinar cells (arrowhead) and duct lumen (arrow); (O) Omission of the primary antibody (No 1°) as a negative control. This was an adjacent section to that shown in panel (I) focusing on the region of inflammation where specific staining was intense when the primary antibody was included. The scale bar in (J) applies to (A-J). The scale bar in (O) applies to (K-O).

Immunohistochemical staining for Reg3α was used to visualize expression patterns in wild type and CF mice, under control conditions and during pancreatitis. The wild type control pancreas had little staining (Fig. 5A). When pancreatitis was induced, staining for Reg3α was variable and there were areas of strong reactivity in the tissue at different times (Fig. 5B–D). Similar foci of Reg3α immunoreactivity have been previously noted in areas of inflammation in caerulein-induced experimental pancreatitis in rats [1]. A region of strong immunoreactivity in a wild type pancreas at 1 h after a single caerulein injection (Fig. 5B) is shown at higher magnification in Fig. 5K. The strongly labeled cells are in a highly inflamed area with many infiltrated leukocytes. Interestingly, Reg3α staining was occasionally observed in forming fibrotic tissue (Fig. 5D, L; wild type 3d after caerulein treatment). Immunoreactivity was frequently observed in ductal lumina (arrows in Fig. 5D; wild type, 3d). This observation is consistent with the fact that Reg/PAP are acinar cell secretory proteins [10]. By 7d post-caerulein, the wild type tissue has largely recovered and Reg3α labeling is absent (Fig. 5E).

In contrast to the wild type control, the untreated CF pancreas was strongly immunoreactive for Reg3α with labeling of most acinar cells but not islets (Fig. 5F). Intense labeling was often observed in the dilated acinar luminal spaces (Fig. 5F, M) which are characteristic of this mouse model of CF [14]. During pancreatitis in CF mice, Reg3α staining was variable and there were some strongly stained regions adjacent to weakly stained areas (Fig. 5G; 1 h) or more uniformly labeled tissue (Fig. 5H; 7 h). At higher magnification, the intense labeling can be observed in the apical portion of acinar cells as well as in a ductal lumen (Fig. 5N; arrow, CF 1 h). Similar to wild type mice, intense immunoreactivity in CF mice with pancreatitis was frequently observed in inflamed areas (Fig. 5I; CF, 3d). By 7d after caerulein administration, the CF tissue again had a fairly homogeneous labeling of acinar cells but not islets (Fig. 5J). The antibody staining was specific as shown by omission of the primary antibody (Fig. 5O; CF, 3d); this control sample was from a section adjacent to that shown in Fig. 5I, which was intensely stained when the specific antibody was included.

## Discussion

It is widely believed that overexpression of *Reg/PAP *genes constitutes a protective mechanism in response to pancreatic injury or stress [1,33,34]. While it is clear that the *Reg/PAP *genes are upregulated in response to pancreatic damage in pancreatitis, it is still uncertain what they do in pancreatitis. The most direct evidence comes from an antisense approach which indicates that they can limit the severity of experimental pancreatitis [35]. Using the sodium taurocholate pancreatic ductal infusion model of pancreatitis in rats and an antisense oligonucleotide that targets *PAP1, PAP2, and PAP3 *mRNAs, it was shown that the severity of pancreatitis was increased. In the antisense treated rats with pancreatitis there was 3.5-fold greater serum amylase, increased tissue edema and histological inflammatory scores, and elevated levels of *IL-1α*, *IL-1β*, and *IL-4 *mRNAs in peripheral blood monocytes compared to rats treated with a sense oligonucleotide [35]. The antisense oligonucleotide, but not control sense oligonucleotide, inhibited the induction of the *PAP *genes during pancreatitis by 40–60%. Thus, a modest inhibition of *PAP *gene upregulation during taurocholate-induced pancreatitis increased the severity of the disease response demonstrating the beneficial effect of these genes during pancreatic stress.

These genes are also known to be expressed in tissues other than the pancreas, and in many cases they were shown to be mitogenic or anti-apoptotic, and they promote cell survival in normal development as well as in tissue injury [5-7,36,37]. Their role in inhibiting apoptosis is especially interesting in relation to pancreatitis, as evidence indicates that apoptotic cell death is related to less severe inflammation during pancreatitis as compared to necrotic cell death [8,9].

We previously showed that the CF mouse pancreas overexpresses *Reg3α*, *PAP/Reg3β*, and *Reg3γ *[14]. In general the *Reg/PAP *genes are coordinately upregulated by stress to the pancreas [1] but this does not always hold as shown by microarray analysis of the CF mouse pancreas. Two of the five members of the *Reg/PAP *family genes represented on the microarray, *Reg1/PSP *and *Reg2/PTP *(pancreatic thread protein), were not overexpressed [14].

In a recent study, it was shown that several cytokines and inflammatory markers are upregulated in the CF mouse pancreas, including *ICAM-1*, *TNF-α*, *IL-6*, and *IL-1β *[9]. *PAP *expression in AR42J cells can be activated by TNF-α [38] and it has been shown that the promoter of the *PAP *gene can drive expression of a reporter construct through IL-6 stimulation [39]. The elevated cytokine expression may explain the constitutive overexpression of the *Reg/PAP *genes in the CF mouse pancreas.

We postulated that the CF mouse with already elevated *Reg/PAP *expression would have less severe caerulein-induced pancreatitis than wild type mice. Even so, the time course of pancreatitis was similar comparing CF and wild type mice. It should be noted, however, that the CF pancreatic tissue content of amylase and other zymogens is about 50–75% that of wild type [9,24,40]. Accordingly, if the serum amylase levels were normalized to tissue content, the control CF mice would already have elevated serum amylase compared to wild type, and would also have greater pancreatitis-associated serum amylase levels. In a recent report, it was shown that supramaximal caerulein induced a greater degree of acute pancreatitis in CF mice compared to wild type, based on histological scores, edema, and pancreatic MPO levels [9]. They used 12 hourly injections of caerulein which is more harsh than the 7 injections we used in our study and this might explain the pronounced difference in severity of pancreatitis compared to our results.

The lack of protection from pancreatitis by constitutive overexpression of *Reg/PAP *genes in the CF mouse pancreas is conceivably due to multiple factors. First, there is already a mild inflammation of the tissue [9,14] which may predispose to a more severe pancreatitis and could counteract the protective effect of Reg/PAP. Second, the fact that not all the *Reg/PAP *genes were constitutively elevated in the CF tissue may indicate that the additional overexpression of *Reg1/PSP *and *Reg2/PTP *is needed. It has been shown that caerulein-induced pancreatitis protects against death in animals subsequently treated to induce another experimental model of pancreatitis, infusion of the pancreatic duct with taurocholate [41]. Because *PAP *expression was increased by the caerulein treatment, it was suggested that PAP could mediate the protective effect observed. It is not clear from that study if all the *Reg/PAP *genes were upregulated and if overexpression of a subset of the genes such as occurs in the CF mouse would be protective.

A third possibility is that the time course of *Reg/PAP *gene expression may be of importance in their ability to protect. There is some confusion in the literature on the time course of Reg/PAP gene expression in pancreatitis. A previous study showed a transient downregulation of *PAP/Reg3β *[32] and our current results confirm this result and extend it to *Reg3α *and *Reg3γ*. There is a significant decrease in mRNAs for these genes at very early times after caerulein injection in both wild type and CF mice, followed by recovery and overexpression at later times as the tissue recovers from pancreatitis. These data are in apparent contradiction with published work on *Reg/PAP *gene expression in pancreatitis. Although the *Reg/PAP *genes have been referred to as 'acute phase' genes, the majority of studies have measured their expression levels at 12–24 hours or later after induction of acute pancreatitis [2]. The transient downregulation of the *Reg/PAP *genes in mice with caerulein-induced pancreatitis occurs during the first hours, followed by a rebound and later overexpression. Thus, although these genes are overexpressed in caerulein-induced pancreatitis, they are first transiently downregulated at short times after the insult. It should be noted that the short times have been analyzed only using mice and not rats, and it is conceivable that there will be species differences. The disparity between our results which show a transient decrease in *Reg/PAP *gene expression in the mouse, and those in the literature showing only the later increased expression during pancreatitis in rats, is likely only an apparent one. The differences are probably due to the different time points evaluated and maybe also reflect species variations.

A fourth possibility for failure to protect the pancreas may lie in the idea that apoptotic cell death in pancreatitis results in less severe damage compared to necrotic cell death [8] and that the *Reg/PAP *genes have been demonstrated to be anti-apoptotic [5-7]. It may be that these gene products inhibit programmed cell death of damaged acinar cells in the CF pancreas and result in greater necrotic death during pancreatitis and greater damage. This idea is supported by the recent report of DiMagno et al., who showed decreased apoptosis and more severe inflammation in caerulein-induced pancreatitis in CF mice [9].

Despite the finding that *Reg3α *was highly expressed in the control CF mouse pancreas and that protein levels did not change dramatically during pancreatitis, there were differences in the expression pattern by immunohistochemistry. In the control CF pancreas areas of intense immunoreactivity were associated with areas containing dilated acinar lumina which are characteristic of this mouse model of CF [17]. In addition, similar to wild type mice, foci of intense Reg3α immunoreactivity in the CF mouse pancreas occurred in regions of inflammation and leukocyte infiltration. So, although the level of protein expression did not change dramatically, the localization of intense expression was associated with the more affected areas of the tissue. This finding is consistent with the connection of Reg/PAP expression and damage to cells of the pancreas. Whether focal increases in expression have a positive or negative effect on limiting damage or the rate of recovery remains an open question.

## Conclusion

In summary, the CF mouse exhibits a similar severity of caerulein-induced pancreatitis compared to wild type despite the constitutive high expression of three of the *Reg/PAP *genes. The constitutive upregulation of *Reg/PAP *expression in the CF mouse pancreas may result in balanced protective (anti-inflammatory) and aggressive (anti-apoptotic) factors which could account for the lack of effect on the severity of pancreatitis.

## Abbreviations

cAMP, cyclic AMP; CF, cystic fibrosis; CFTR, cystic fibrosis transmembrane conductance regulator; ELISA, enzyme-linked immunosorbent assay; ICAM-1, intercellular adhesion molecule 1; IL-1β, interleukin 1β; IL-6, interleukin 6; MPO, myeloperoxidase; PAP, pancreatitis associated protein; PSP, pancreatic stone protein; PTP, pancreatic thread protein; Reg, regenerating; TNF-α, tumor necrosis factor α

## Competing interests

The author(s) declare that they have no competing interests.

## Authors' contributions

ON performed the biochemical and PCR determinations. RG and PA performed the ELISAs and immunohistochemistry. RCD designed the studies, analyzed the data, and prepared the manuscript with input from the other authors. All authors have given final approval of the version to be published.

## Pre-publication history

The pre-publication history for this paper can be accessed here:
